# Determination of Oxidized Phosphatidylcholines by Hydrophilic Interaction Liquid Chromatography Coupled to Fourier Transform Mass Spectrometry

**DOI:** 10.3390/ijms16048351

**Published:** 2015-04-14

**Authors:** Pia Sala, Sandra Pötz, Martina Brunner, Martin Trötzmüller, Alexander Fauland, Alexander Triebl, Jürgen Hartler, Ernst Lankmayr, Harald C. Köfeler

**Affiliations:** 1 Core Facility for Mass Spectrometry, Medical University of Graz, Stiftingtalstrasse 24, Graz 8010, Austria; E-Mails: pia.sala@tu-dresden.de (P.S.); sandra.poetz@pccl.at (S.P.); martina.brunner@medunigraz.at (M.B.); martin.troetzmueller@medunigraz.at (M.T.); alexander.fauland@ki.se (A.F.); alexander.triebl@medunigraz.at (A.T.); 2 Institute of Analytical Chemistry and Food Chemistry, Graz University of Technology, Stremayrgasse 9/II, Graz 8010, Austria; E-Mail: lankmayr@tugraz.at; 3 Bioinformatics Group, Institute for Knowledge Discovery, Graz University of Technology, Petersgasse 14, Graz 8010, Austria; E-Mail: juergen.hartler@tugraz.at; 4 Omics Center Graz, Stiftingtalstrasse 24, Graz 8010, Austria

**Keywords:** fourier transform orbitrap mass spectrometry, hydrophilic interaction liquid chromatography, oxidized low density lipoprotein, oxidized phospholipids, LC-MS

## Abstract

A novel liquid chromatography-mass spectrometry (LC-MS) approach for analysis of oxidized phosphatidylcholines by an Orbitrap Fourier Transform mass spectrometer in positive electrospray ionization (ESI) coupled to hydrophilic interaction liquid chromatography (HILIC) was developed. This method depends on three selectivity criteria for separation and identification: retention time, exact mass at a resolution of 100,000 and collision induced dissociation (CID) fragment spectra in a linear ion trap. The process of chromatography development showed the best separation properties with a silica-based Kinetex column. This type of chromatography was able to separate all major lipid classes expected in mammalian samples, yielding increased sensitivity of oxidized phosphatidylcholines over reversed phase chromatography. Identification of molecular species was achieved by exact mass on intact molecular ions and CID tandem mass spectra containing characteristic fragments. Due to a lack of commercially available standards, method development was performed with copper induced oxidation products of palmitoyl-arachidonoyl-phosphatidylcholine, which resulted in a plethora of lipid species oxidized at the arachidonoyl moiety. Validation of the method was done with copper oxidized human low-density lipoprotein (LDL) prepared by ultracentrifugation. In these LDL samples we could identify 46 oxidized molecular phosphatidylcholine species out of 99 possible candidates.

## 1. Introduction

Non-enzymatic cellular oxidation processes are triggered by reactive oxygen species (ROS) such as hydrogen peroxide, superoxide anions or peroxide anions produced in the respiratory chain in mitochondria. Under normal cellular conditions ROS are immediately eliminated by various antioxidants, but in case of an imbalance in the antioxidant system excessive ROS may react with a wide variety of biomolecules and pave the way to certain pathophysiological conditions [1]. One compound class highly susceptible to ROS are polyunsaturated fatty acids (PUFA), particularly when esterified to membrane phospholipids in close proximity to ROS production. This lipid peroxidation process preferably starts at *bis*-allylic methylene groups in PUFA and leads to a plethora of molecular phospholipid oxidation products, containing hydroxy, hydroperoxy, ketene, aldehyde and carboxylic acid groups [2,3]. Furthermore, truncation of the esterified PUFA at various positions adds another layer of molecular complexity. Due to their wide variety of chemical functionalities, oxidized phospholipids do not cause a singular effect in cellular systems but act in a plethora of mechanisms, sometimes even contradictory in the physiological sense [4]. Therefore, oxidized phospholipids are involved in many different pathophysiological processes, resulting in diseases like atherosclerosis, acute inflammation, ischemia and multiple sclerosis, to name just a few [5,6].

Since only minute amounts of oxidized lipid species are to be expected in biological systems, analysis of oxidized phospholipids is a challenging task. Due to its high sensitivity and selectivity, mass spectrometry coupled to liquid chromatography is a suitable technology for this challenge [7]. At the chromatography end, most groups work with reversed phase HPLC relying on separation by fatty acyl hydrophobicity [8,9,10], while only a few use normal phase separation [11,12] or a combination of both polarities in a 2D approach [13]. The advantage of recently emerging hydrophilic interaction liquid chromatography (HILIC) separation techniques in lipid analysis is the ability to separate phospholipids by their polar head groups rather than by their non-polar fatty acyl moieties [14,15,16], which is important for lipids with multiple functional groups like oxidized phospholipids. Another substantial advantage is “pseudo normal phase separation” using aqueous solvent mixtures compatible with the electrospray ionization (ESI) process. At the mass spectrometry end, most conventional platforms use low resolution instrumentation (ion trap, triple quadrupole) relying on retention time and characteristic fragments [8,9,10,13]. Nevertheless, the analytical power for determination of oxidized phospholipids can be tremendously increased by the use of high resolution equipment [17,18], even more so by Fourier transform-mass spectrometry (FT-MS) instruments based either on ion cyclotron resonance or on Orbitrap technology [19,20,21,22].

We matched the challenges of oxidized phospholipid analysis by development of a liquid chromatography-mass spectrometry (LC-MS) method based on HILIC separation, high resolution Orbitrap mass spectrometry on intact molecular ions and by automated data dependent linear ion trap fragment spectra. The instrumental setting is complemented by data processing with a Lipid Data Analyzer, a customized in-house developed software solution [23].

## 2. Results and Discussion

### 2.1. HPLC and Mass Spectrometry Development

**Figure 1:**
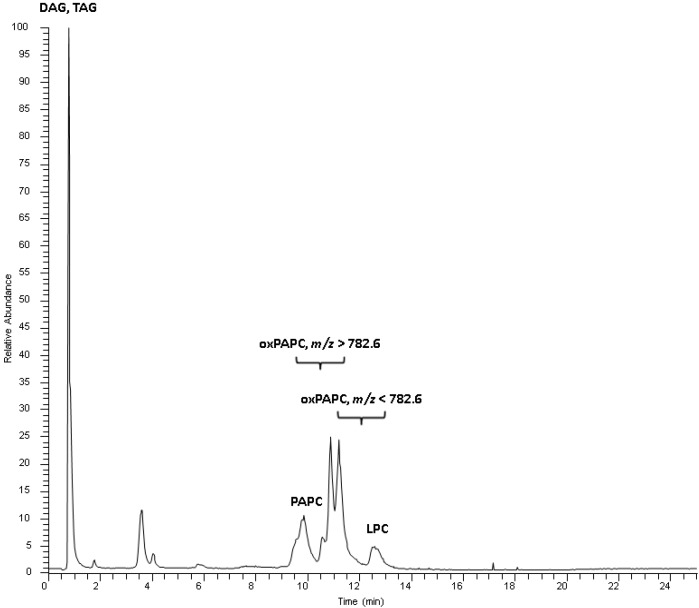
Total ion chromatogram of a standard mix containing oxidized and non-oxidized PAPC. Oxidized PAPC species elute according to polarity and carbon number of the oxidized arachidonic acid moiety between the non-oxidized PAPC and lysophosphatidylcholines (LPC). Triacylglycerols (TAG) and diacylglycerols (DAG) elute with the void volume.

**Figure 2:**
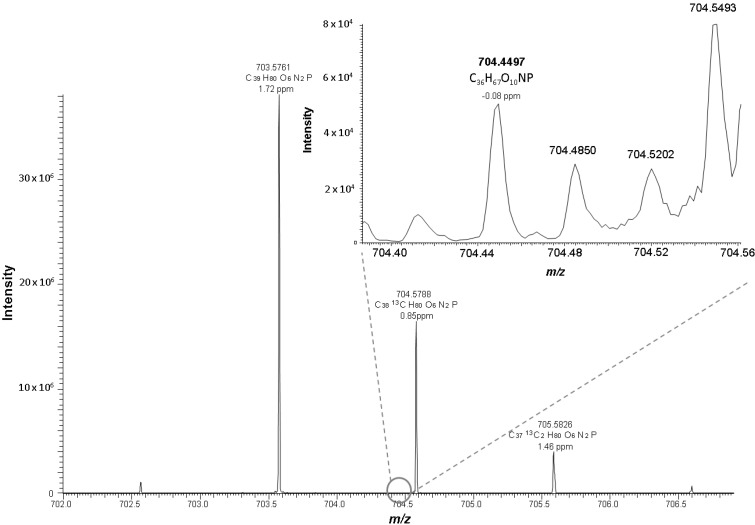
Positive ESI mass spectrum of oxidized LDL at the retention time 11.38 min. In the depicted spectrum PC 18:0_10:3[2O] at *m*/*z* 704.4497 co-elutes at the same retention time as SM 16:0 and has the same nominal mass as the M+1 peak of SM 16:0 (*m*/*z* 704.5788).

One major aim of this study was the development of a method for separation of oxidized phosphatidylcholines from non-oxidized, as well as from other, lipid classes. The challenge of this endeavor was on the one hand, the similar polarity of some oxidized and native phospholipids, and on the other hand the big range of oxidized species polarity to be covered. We focused just on phosphatidylcholine, since it is the most abundant phospholipid class in biological membranes and consequently most oxidized phospholipids are to be expected from this lipid class. For the sake of simplicity we used only oxidized 1-palmitoyl-2-arachidonyl-phosphatidylcholine (PAPC; PC 16:0/20:4) for chromatographic method development. Oxidation of PAPC results in an ample array of oxidized products ranging from truncated species with only five carbon atoms at *sn*-2 position to complex rearrangements of arachidonic acid to isoprostaglandins, isolevuglandins and isothromboxanes [2]. This artificial mix fulfilled our demands for a wide range of polarities and functional groups well, and we could identify 36 oxidized species originating from PAPC (Supplementary Table S1). Additionally to these oxidized PAPC species, we used a set of defined lipid standards (Supplementary Table S2) for development of chromatography. We started chromatographic development with an YMC Diol HILIC column but finally switched to a Kinetex HILIC column since this column resulted in better chromatographic resolution. Due to the huge range of oxidized *sn*-2 fatty acyl chain lengths and functional groups it was not possible to elute all oxidized PAPC species in just one defined chromatographic peak as we initially aimed for, but nevertheless oxidized species are sufficiently separated from the non-oxidized species (Figure 1) to avoid the adverse effects of ion suppression of co-eluting compounds as is the case in all shotgun methods [20,21,22]. Furthermore we were able to add another layer of separation by high resolution mass spectrometry (R = 100,000 at *m*/*z* 400), for detecting lipid species which are unresolved by chromatography, thereby resulting in confident information about elemental compositions by exact mass on molecular ions. Figure 2 exemplifies the advantages of our high resolution mass spectrometry platform by detection of a minor oxidized phospholipid compound (PC 18:0_10:3[2O]) at the same nominal mass and retention time as a major lipid (M+1 peak of *N*-palmitoyl-sphingomyelin). The maximum mass resolution needed for separation of oxygenated (C_n-1_H_m-4_O_9_NP) from non-oxygenated (C_n_H_m_O_8_NP) phosphatidylcholine (PC) or phosphatidylethanolamine (PE) compounds would be at least 22,000 at *m*/*z* 800, which is easily achieved by Orbitrap mass spectrometry. This is clearly an advantage over low-resolution instrumentation, and provides a high degree of certainty even without collision induced activation (CID) fragmentation such as used in low-resolution single reaction monitoring (SRM) methods [8,10,20]. 

### 2.2. Identification Strategy for Oxidized Phospholipids

Unambiguous structural identification of lipids in general and oxidized phospholipids in particular is hardly achieved with conventional LC-MS or matrix assisted laser desorption ionization-time of flight (MALDI-TOF) equipment, because low energy CID or post source decay (PSD) spectra rarely cover in depth structural features like stereochemistry. For a deeper coverage of structural details like double bond position or formation of ring structures, one would need high energy CID (four sector MS, MALDI-TOF/TOF) or electron impact ionization, both enabling charge remote fragmentations [24,25], or ozonolysis for location of double bonds [26]. Additionally, nuclear magnetic resonance spectroscopy could support in depth stereochemical structural elucidation [12]. The trade-off of the aforementioned methods is often a compromised sensitivity and/or missing chromatography. 

Bearing this in mind we identified oxidized phosphatidylcholines as comprehensively as possible at uncompromised sensitivity and applicability of the method in terms of acquisition speed. The aim of establishing the heuristic scoring system depicted in Table 1 is to provide an estimation of identification certainty at a glance. The scoring system relies on exact mass of the molecular precursor and specific fragments in low resolution linear ion trap CID spectra. In a first step, we evaluated whether the elemental composition displayed in each extracted 3D ion chromatogram would fit with any oxidized phosphatidylcholine species. To this end, we generated a mass list of all literature of known oxidized phosphatidylcholine species and some additional likely elemental compositions and identified all matching mass peaks at a mass tolerance of 5 ppm by Lipid Data Analyzer software. Out of these candidates, only the ones with less than 2 ppm mass deviation gained a scoring point. In a second step, we took MS/MS spectra from data dependent analysis into account. If the top 10 methods did not either trigger MS/MS on a precursor, or only (mixed) MS/MS spectra of too low quality were available, the candidate was rejected and resulted in just one scoring point. If MS/MS spectra of sufficient quality were available, a scoring point was added for head group specific fragments indicative for PC. These fragments were either the choline phosphate head group at *m*/*z* 184 or the neutral loss of 59 Da. Therefore identification as oxidized PC species according to the described scoring system was achieved if at least two scoring points were assigned. The next two scoring points were assigned if the neutral losses of the presumably oxidized fatty acid moiety were detected, one for the fatty acyl (R_ox_-COOH) loss and another one for the corresponding ketene (R_ox_-C=O) loss. At this stage, it was possible to assign fatty acids in the molecular structure. Additionally 0.5 scoring points were assigned for a neutral loss of one or two H_2_O. This neutral loss is considered to be non-specific, which is reflected by only half the scoring weight of 0.5 instead of 1.0 points in the scoring system. Nevertheless, unmodified PC compounds do not show any H_2_O loss and therefore these fragments could indicate additional hydroxy groups or rearranged carbonyl functionalities, which corroborates the identity of an oxidized PC. We did not take into account retention times in our scoring system because chemically pure standard compounds would have been required for each oxidized PC species. Since only a few oxidized PC species are commercially available, this was not an option. Despite these constraints, we ensured that retention time and proposed structure would approximately fit together in terms of polarity.

**Table 1:** Presented scoring scheme to estimate the reliability of a hit.

Requirement	Fragment	Scoring Points
Exact mass < 2 ppm		1.0
Headgroup fragment	[PChol]^+^ or [M-59]^+^	1.0
Fatty acyl fragment	[M-R_ox_]^+^	1.0
Fatty acyl fragment	[M-R_ox_=C=O]^+^	1.0
Water loss at oxidized moiety	[M-H_2_O]^+^	0.5
Water loss at oxidized moiety	[M-2H_2_O]^+^	0.5

### 2.3. Analysis of Oxidized Phosphatidylcholine Species in Oxidized Low Density Lipoprotein 

Finally, we evaluated our method with Cu^2+^ oxidized low-density lipoprotein (LDL). The Lipid Data Analyzer (LDA) identified 99 possible oxidized PC candidates by exact mass and isotopic pattern in 3D mass chromatograms (*m*/*z*, retention time, and intensity). Even though LDA would have supported retention time constraints [23], they were not set in this analysis since the retention time of the compounds is not exactly known. Out of these candidates, 46 compounds could be identified unambiguously as oxidized PC compounds, and 28 of them could be assigned with fatty acid composition (Table 2). Finally, we matched the species in the higher scoring ranks with known possible structures from the literature to assign potential candidate structures for the detected lipid species annotated in shorthand nomenclature (Table 3) [27]. In this sense, our platform provides a good starting point for further unambiguous structural in-depth confirmation of the identified oxidized PC species. In comparison to our existing reversed phase method [28], the HILIC method delivered a deeper coverage of oxidized PCs (46 *vs*. 22 species). This might be due to better chromatographic separation and subsequently less ionization suppression effects. These results compare well to existing results [8,9], but certainty is boosted by exact mass on molecular ions. The relative contributions to the total pool of oxidized PC (Table 2) revealed linoleic acid containing PC species to be the main precursors of oxidized PC. Furthermore the majority (73.8%) of oxidized PC species were non-truncated oxygenated compounds. This indicates a rather mild oxidation process including some rearrangements in the PUFA, but rather few C-C bond cleavages.

**Table 2:** List of observed exact masses, elemental compositions, retention times (RT) and corresponding compound assignments by shorthand nomenclature in oxidized LDL. Relative contribution is calculated by peak areas of mass chromatograms where the sum of all oxidized PC species detected equals 100%. Score is calculated according to the scheme in Table 1.

Species	Elemental Composition	*m*/*z* Observed [M+H]^+^	Δ ppm	RT [min]	Relative Contribution [%]	Score
PC 16:0_20:4 [4O]	C_44_H_80_O_12_NP	846.5502	1.30	10.23	0.91	5.0
PC 16:0_20:3 [4O]	C_44_H_82_O_12_NP	848.5655	0.93	10.38	0.71	5.0
PC 18:0_20:5 [2O]	C_46_H_82_O_10_NP	840.5750	0.15	10.09	0.54	5.0
PC 18:0_20:4 [3O]	C_46_H_84_O_11_NP	858.5850	0.57	9.64	3.40	5.0
PC 18:0_20:4 [4O]	C_46_H_84_O_12_NP	874.5793	−1.11	10.06	0.83	5.0
PC 16:0_5:1 [O]	C_29_H_56_O_9_NP	594.3770	0.82	11.82	1.48	4.5
PC 16:0_8:3 [3O]	C_32_H_58_O_11_NP	664.3829	1.33	12.20	0.01	4.5
PC 16:0_8:2 [3O]	C_32_H_60_O_11_NP	666.3986	1.33	11.48	0.03	4.5
PC 16:0_22:4 [O]	C_46_H_84_O_9_NP	826.5960	−1.79	12.03	0.56	4.5
PC 16:0_4:1 [O]	C_30_H_58_O_9_NP	608.3920	0.30	11.82	0.60	4.5
PC 16:0_6:1 [O]	C_33_H_62_O_10_NP	664.4178	0.73	11.08	0.09	4.5
PC 16:0_9:2 [2O]	C_46_H_84_O_9_NP	826.5960	0.52	9.65	0.16	4.5
PC 16:0_9:0 [O]	C_33_H_64_O_9_NP	650.4402	−1.69	10.86	7.38	4.0
PC 16:0_8:2 [2O]	C_32_H_60_O_10_NP	650.4036	1.43	11.22	0.13	4.0
PC 16:0_20:6 [2O]	C_44_H_76_O_10_NP	810.5290	1.24	10.23	1.71	4.0
PC 16:0_20:5 [3O]	C_44_H_78_O_11_NP	828.5393	0.91	10.06	3.28	4.0
PC 16:0_20:4 [3O]	C_44_H_80_O_11_NP	830.5552	1.27	9.81	6.08	4.0
PC 16:0_20:3 [3O]	C_44_H_82_O_11_NP	832.5687	−1.25	10.01	3.02	4.0
PC 16:0_22:6 [2O]	C_46_H_80_O_10_NP	838.5590	0.29	10.06	1.41	4.0
PC 16:0_12:2 [2O]	C_36_H_68_O_10_NP	706.4650	0.52	10.97	0.06	3.5
PC 16:0_18:2 [2O]	C_42_H_80_O_10_NP	790.5577	1.85	10.04	15.86	3.5
PC 18:0_9:0 [O]	C_35_H_68_O_9_NP	678.4714	−1.53	10.68	5.08	3.5
PC 18:0_18:2 [O]	C_44_H_84_O_9_NP	802.5946	1.22	9.81	6.12	3.5
PC 16:0_20:4 [O]	C_44_H_80_O_9_NP	798.5651	1.03	9.81	0.94	3.5
PC 18:0_5:1 [O]	C_31_H_60_O_9_NP	622.4080	−0.29	11.62	1.90	3.5
PC 18:0_5:1 [2O]	C_31_H_60_O_10_NP	638.4027	0.00	10.74	0.20	3.5
PC 16:0_10:3 [2O]	C_34_H_62_O_10_NP	676.4193	−1.44	11.10	0.08	3.5
PC 18:0_10:3 [2O]	C_36_H_66_O_10_NP	704.4497	−0.08	10.49	0.18	3.5
PC 36:5 [2O]	C_44_H_78_O_10_NP	812.5440	−0.53	10.23	1.57	3.0
PC 38:6 [2O]	C_46_H_80_O_10_NP	838.5590	−0.29	10.10	1.61	3.0
PC 40:6 [O]	C_48_H_84_O_9_NP	850.5960	−0.50	10.70	0.10	2.5
PC 34:2 [O]	C_42_H_80_O_9_NP	774.5643	−0.08	9.98	20.03	2.5
PC 27:0 [2O]	C_35_H_68_O_10_NP	694.4650	0.53	10.87	1.30	2.5
PC 36:4 [2O]	C_44_H_80_O_10_NP	814.5590	0.30	9.75	5.02	2.5
PC 26:3 [2O]	C_34_H_62_O_10_NP	676.4183	0.00	10.86	0.08	2.5
PC 26:3 [3O]	C_34_H_62_O_11_NP	692.4130	0.44	12.75	0.03	2.5
PC 26:2 [3O]	C_34_H_64_O_11_NP	694.4284	0.70	10.94	0.06	2.5
PC 20:1 [2O]	C_28_H_54_O_10_NP	596.3548	1.64	11.73	0.03	2.5
PC 22:1 [2O]	C_30_H_58_O_10_NP	624.3871	0.00	11.56	0.09	2.5
PC 27:3 [O]	C_35_H_64_O_9_NP	674.4381	1.45	11.75	0.07	2.5
PC 36:6 [O]	C_44_H_76_O_9_NP	794.5325	0.62	11.24	0.11	2.5
PC 25:0 [2O]	C_33_H_64_O_10_NP	666.4351	−1.56	10.55	1.31	2.0
PC 28:3 [3O]	C_36_H_66_O_11_NP	720.4446	0.00	10.38	0.06	2.0
PC 21:1 [2O]	C_29_H_56_O_10_NP	610.3720	0.91	12.43	0.11	2.0
PC 34:6 [O]	C_42_H_72_O_9_NP	766.5020	−0.40	12.74	0.03	2.0
PC 24:2 [O]	C_32_H_60_O_9_NP	634.4088	−1.64	11.45	0.06	2.0

**Table 3:** List of detected fragments in oxidized LDL including assignment of possible non-oxidized precursors and oxidized PC structures reported in the literature for the corresponding shorthand denoted compounds. Abbreviations of oxidized structures are explained in Supplementary Table S3.

Species	Possible Oxidized Structure	Possible Precursor	[PChol]^+^ [M-59]^+^	[M-R_ox_]^+^ [M-R_ox_=C=O]^+^	[M-H_2_O]^+^ [M-2H_2_O]^+^	Score
PC 16:0_20:4 [4O]	isoPGG2	PC 16:0/20:4	787	496, 478	828, 810	5.0
PC 16:0_20:3 [4O]	isoTxA2	PC 16:0/20:4	789	496, 478	830, 812	5.0
PC 18:0_20:5 [2O]	isoPGJ2/A2	PC 18:0/20:4	781	524, 506	822, 804	5.0
PC 18:0_20:4 [3O]	isoPGE_2_/D_2_ isoLGE_2_/D_2_ isoTXB_2_	PC 18:0/20:4	799	524, 506	840, 822	5.0
PC 18:0_20:4 [4O]	isoPGG2	PC 18:0/20:4	815	524, 506	856, 838	5.0
PC 16:0_5:1 [O]	OV	PC 16:0/20:4	184	496, 478	576	4.5
PC 16:0_8:3 [3O]	KOdiA	PC 16:0/20:4	184	496, 478	646	4.5
PC 16:0_8:2 [3O]	HOdiA	PC 16:0/20:4	184	496, 478	648	4.5
PC 16:0_22:4 [O]		PC 16:0/22:4	767	496, 478	808	4.5
PC 16:0_4:1 [O]		PC 16:0/22:6	184	496, 478	562	4.5
PC 16:0_6:1 [O]		PC 18:0/22:6	184	496, 478	590	4.5
PC 16:0_9:2 [2O]		PC 18:0/22:6	184	496, 478	646	4.5
PC 16:0_9:0 [O]	ON	PC 16:0/18:2	184	496, 478		4.0
PC 16:0_8:2 [2O]	HOOA	PC 16:0/20:4	184	496, 478		4.0
PC 16:0_20:6 [2O]	EC	PC 16:0/20:4	184	496	792, 774	4.0
PC 16:0_20:5 [3O]	EI	PC 16:0/20:4	769	496	810, 792	4.0
PC 16:0_20:4 [3O]	isoPGE_2_/D_2_ isoLGE_2_/D_2_ isoTXB_2_	PC 16:0/20:4	771	496	812, 794	4.0
PC 16:0_20:3 [3O]	isoPGF2α	PC 16:0/20:4	773	496	814, 796	4.0
PC 16:0_22:6 [2O]		PC 16:0/22:6	779	496	820, 802	4.0
PC 16:0_12:2 [2O]	HODA	PC 16:0/18:2	184	478	688	3.5
PC 16:0_18:2 [2O]	HpODE	PC 16:0/18:2	184	496	772	3.5
PC 18:0_9:0 [O]	ON	PC 18:0/18:2	184	524	660	3.5
PC 18:0_18:2 [O]	HODE	PC 18:0/18:2	184	524	784	3.5
PC 16:0_20:4 [O]	HETE	PC 16:0/20:4	184	478	680	3.5
PC 18:0_5:1 [O]	OV	PC 18:0/20:4	184	524	604	3.5
PC 18:0_5:1 [2O]	G	PC 18:0/20:4	184	506	620	3.5
PC 16:0_10:3 [2O]		PC 16:0/22:6	184	496	658	3.5
PC 18:0_10:3 [2O]		PC 18:0/22:6	184	506	686	3.5
PC 36:5 [2O]		PC 16:0/20:4	753		794, 772	3.0
PC 38:6 [2O]		PC 18:0/20:4	779		820, 802	3.0
PC 40:6 [O]		PC 18:0/22:6	791		832	2.5
PC 34:2 [O]		PC 16:0/18:2	184		756	2.5
PC 27:0 [2O]		PC 18:0/18:2	184		676	2.5
PC 36:4 [2O]		PC 16:0/20:4	755		796	2.5
PC 26:3 [2O]		PC 18:0/20:4	184		658	2.5
PC 26:3 [3O]		PC 18:0/20:4	184		674	2.5
PC 26:2 [3O]		PC 18:0/20:4	184		676	2.5
PC 20:1 [2O]		PC 16:0/22:6	184		578	2.5
PC 22:1 [2O]		PC 18:0/22:6	184		606	2.5
PC 27:3 [O]		PC 18:0/22:6	184		656	2.5
PC 36:6 [O]		PC 18:0/22:6	184		776	2.5
PC 25:0 [2O]		PC 16:0/18:2	184			2.0
PC 28:3 [3O]		PC 16:0/18:2	184			2.0
PC 21:1 [2O]		PC 16:0/20:4	184			2.0
PC 34:6 [O]		PC 16:0/22:6	184			2.0
PC 24:2 [O]		PC 18:0/22:6	184			2.0

In summary, we developed a HILIC-coupled high-resolution LTQ-Orbitrap method for the analysis of oxidized PC species including data-dependent MS/MS spectra. This method enabled us to identify 46 oxidized PC species by exact mass of molecular ions and selective fragments in oxidized LDL. 

## 3. Material and Methods 

### 3.1. Materials

All lipid standards were purchased from Larodan (Malmö, Sweden). A complete list is shown in Supplementary Table S1. Standard stock solutions were dissolved in chloroform/methanol 1:1 (*v*/*v*) at a concentration of 1 mM and stored at −20 °C. Lipid standard mixtures were prepared freshly every day in chloroform/methanol 1:1 (*v*/*v*) at a concentration of 3 µM and were used immediately. 

Chloroform was HPLC grade, ammonium acetate and formic acid were analytical grade, all obtained from Merck KGaA (Darmstadt, Germany). 2-Propanol was LC-MS grade and supplied by Fluka (Steinheim, Germany). Methanol and acetonitrile (LC/MS grade), and 28% ammonia p.a. were all purchased from Sigma-Aldrich Chemie GmbH (Steinheim, Germany). Nitrogen (purity 99.9990) was obtained from Air Liquide (Graz, Austria). Ultra pure water purified by a Milli-Q Gradient system (Millipore, Bedford, MA, USA) was used in all experiments (resistivity > 18 MΩcm). 

### 3.2. Oxidation of PAPC

PAPC was dissolved in 1 mL methanol with a concentration of 3.3 mg/mL. The solution was evacuated in a SpeedVac (Thermo Fisher Scientific, San Jose, CA, USA), mixed with 1 mL of a 5 µM CuSO_4_·5H_2_O–solution in PBS-buffer, vortexed for 5 min and was then sonicated in an ice/water bath for 30 min. After incubation for 113 h at room temperature, the oxidation was quenched by addition of 2 mL chloroform/methanol (2:1) (*v*/*v*) and the resulting suspension was centrifuged at 1360 times g at room temperature for 5 min. The upper aqueous phase was extracted two times with 1 mL chloroform/methanol (2:1) (*v*/*v*) each. The lower organic phases were collected and washed two times with ultra pure water. The solvent of the organic phases was evaporated in a SpeedVac, the residue was re-dissolved in chloroform/methanol (1:1) (*v*/*v*), overlaid with nitrogen (g) and stored at −80 °C until use [29,30].

### 3.3. Isolation and Oxidation of LDL

LDL was isolated from the sera of healthy blood donors by ultracentrifugation in successive KBr gradients as described in detail [21] and stored at 4 °C until use. LDL (1.6 mL) was dialysed overnight in 600 mL phosphate buffered saline (PBS) in an ice/water bath before oxidation was performed. Following that, 250 µL of dialysed LDL were mixed with CuSO_4_·5H_2_O and PBS-puffer and the concentration of CuSO_4_ was adjusted to 40 µM. This mixture was incubated for 25 h at 37 °C in air for oxidation [30]. The reaction was quenched by the addition of 2 mL of chloroform/methanol (2:1) (*v*/*v*) and the lipids were extracted by a Folch extract [29]. After extraction the lipids were diluted in 200 µL chloroform/methanol (1:1) (*v*/*v*) and stored at −80 °C until use.

### 3.4. High Performance Liquid Chromatography-Mass Spectrometry

HILIC-HPLC was performed on an Accela HPLC system (Thermo Scientific, San Jose, CA, USA) equipped with a 100 mm length, 2.1 mm i.d. Kinetex column with 2.6 µm particle size and 100 Å pore size (Phenomenex, Aschaffenburg, Germany). Mobile phase A was water with 10 mM ammonia acetate and 0.5% formic acid. Mobile phase B was acetonitrile/2-propanol 5:2 (*v*/*v*) containing 10 mM ammonia acetate and 0.5% formic acid. The binary gradient started with 95% B decreasing to 80% B in 15 min where it was held for 20 min. Thereafter the column was re-equilibrated to 95% B for 10 min. The total run time was 30 min, at a flow rate of 250 µL/min, with a column oven temperature of 50 °C and a tray temperature of 10 °C. The injection volume was 3 µL. 

A LTQ-Orbitrap hybrid linear ion trap—Orbitrap mass spectrometer (Thermo Fisher Scientific, Bremen, Germany) equipped with an electrospray ion source was used. The instrument was operated in preview mode for parallel MS/MS spectra in the linear ion trap, while running the Orbitrap in full scan mode at 100,000 resolution (*m*/*z* 400) from *m*/*z* 350 to 1000 in positive ESI mode. Helium was used as damping gas. From the FT preview scan, the 10 most abundant ions were selected in data dependent acquisition (DDA), fragmented in the linear ion trap analyzer and detected at nominal mass resolution. The following parameters were used for positive and negative MS/MS experiments: Normalized collision energy was 35%, the repeat count was 2 and the exclusion duration 15 s. The activation Q was at 0.2 and the isolation width 1.2. The spray voltage was set to 5 kV, the tube lens offset was at 120 V and the capillary temperature 250 °C. Data analysis was performed by Lipid Data Analyzer software as described previously [23]. Shorthand annotation of lipid species was done according to LipidMAPS shorthand nomenclature [27]. Since oxidized phospholipids are not covered in the aforementioned publication we amended the existing shorthand nomenclature by putting the number of additional oxygens in parenthesis at the end of the moiety they are attached with. The number behind the colon of the oxidized moiety in this case reflects not only –C=C– double bonds but also ring equivalents and carbonyl groups.

## Supplementary Materials

Supplementary materials can be found at http://www.mdpi.com/1422-0067/16/04/8351/s1.
