# Supplementary material for: Determination of Oxidized Phosphatidylcholines by Hydrophilic Interaction Liquid Chromatography Coupled to Fourier Transform Mass Spectrometry
Source: Int J Mol Sci. 2015 Apr 14;16(4):8351–63. doi: 10.3390/ijms16048351 (PMC4425085; doi:10.3390/ijms16048351)
Supplement: Supplementary file 1 [file ijms-16-08351-s001.pdf]

Supplementary Information

Table S1. List of oxidized species derived by Cu^{2+} oxidation of PAPC.

Possible Oxidized Species	Elemental Composition	Exact Mass [g/mol]	Possible Structure of Oxidized Moiety
PAPC	$C_{44}H_{80}NO_8P$	781.56211	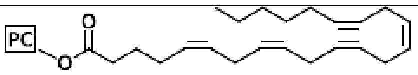
PGPC	$C_{29}H_{56}NO_{10}P$	609.36413	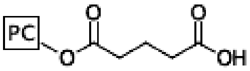
POVPC	$C_{29}H_{56}NO_9P$	593.36922	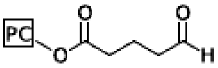
PONPC	$C_{33}H_{64}NO_9P$	694.43182	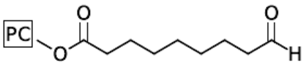
P-HOdiA-PC	$C_{32}H_{60}NO_{11}P$	665.39034	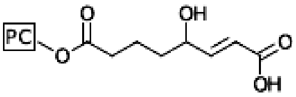
P-KOdiA-PC	$C_{32}H_{58}NO_{11}P$	663.37469	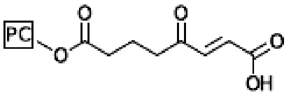
P-HOOA-PC	$C_{32}H_{60}NO_{10}P$	649.39543	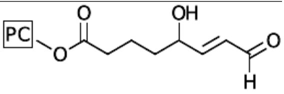
P-KOOA-PC	$C_{32}H_{58}NO_{10}P$	647.37978	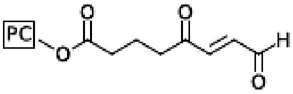
P-HpETE-PC	$C_{44}H_{80}NO_{10}P$	813.55193	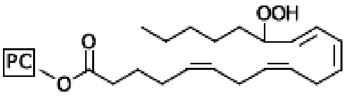
P-HETE-PC	$C_{44}H_{80}NO_9P$	797.55702	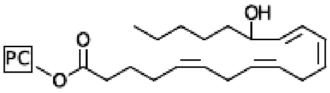
5,6-PEIPC	$C_{44}H_{78}NO_{11}P$	827.53119	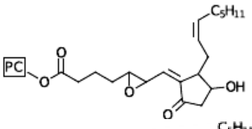
5,6-PECPC	$C_{44}H_{76}NO_{10}P$	809.52063	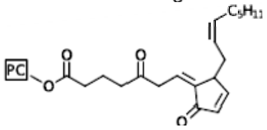
P-isoPGA₂-PC	$C_{44}H_{78}NO_{10}P$	811.53628	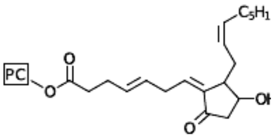
P-isoPGF₂-PC	$C_{44}H_{82}NO_{11}P$	831.56249	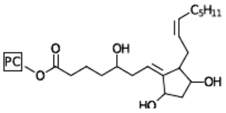
P-isoPGG₂-PC	$C_{44}H_{80}NO_{12}P$	845.54175	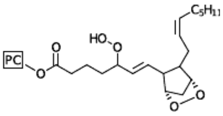
P-KODA-PC	$C_{36}H_{66}NO_{11}P$	719.43729	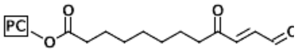
P-HDdiA-PC	$C_{36}H_{68}NO_{11}P$	721.45294	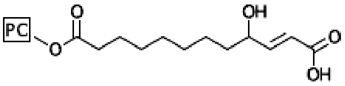

Table S1. *Cont.*

Possible Oxidized Species	Elemental Composition	Exact Mass [g/mol]	Possible Structure Of Oxidized Moiety
P-isoLG-PC	$C_{44}H_{80}NO_{11}P$	829.54684	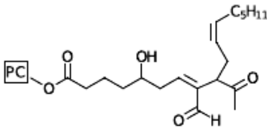
P-isoTxB₂-PC	$C_{44}H_{82}NO_{11}P$	847.55740	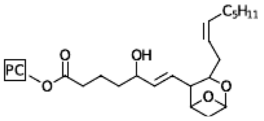
P-furanoyl PC	$C_{32}H_{58}NO_9P$	631.38487	
PNDPC	$C_{33}H_{64}NO_{10}P$	665.42673	
	$C_{31}H_{58}NO_9P$	619.38487	no matching literature known structure
	$C_{31}H_{60}NO_9P$	621.40052	no matching literature known structure
	$C_{32}H_{62}NO_9P$	635.41617	no matching literature known structure
	$C_{33}H_{62}NO_9P$	647.41617	no matching literature known structure
	$C_{33}H_{62}NO_{10}P$	663.41108	no matching literature known structure
	$C_{34}H_{68}NO_9P$	665.46312	no matching literature known structure
	$C_{42}H_{80}NO_9P$	773.55702	no matching literature known structure
	$C_{42}H_{78}NO_{10}P$	787.53628	no matching literature known structure
	$C_{42}H_{78}NO_{11}P$	803.53119	no matching literature known structure
	$C_{42}H_{80}NO_{10}P$	789.55193	no matching literature known structure
	$C_{44}H_{82}NO_9P$	799.57267	no matching literature known structure
	$C_{44}H_{82}NO_{10}P$	815.56758	no matching literature known structure
	$C_{44}H_{84}NO_9P$	801.58832	no matching literature known structure
	$C_{44}H_{84}NO_{10}P$	817.58323	no matching literature known structure
	$C_{46}H_{84}NO_{11}P$	841.58323	no matching literature known structure

Table S2. List of standard compounds used for development of HILIC chromatography.

IUPAC Names of Lipid-Standard Substances	Synonym
1,2-dipalmitoyl-*sn*-glycero-3-phosphocholine	PC 16:0/16:0
1,2-dibehenoyl-*sn*-glycero-3-phosphocholine	PC 22:0/22:0
1,2-dimyristoyl-*sn*-glycero-3-phosphocholine	PC 14:0/14:0
1,2-dilauroyl-*sn*-glycero-3-phosphocholine	PC 12:0/12:0
1,2-distearoyl-*sn*-glycero-3-phosphocholine	PC 18:0/18:0
1,2-dieicosanoyl-*sn*-glycero-3-phosphocholine	PC 20:0/20:0
1-hexadecanoyl-2-(5*Z*,8*Z*,11*Z*,14*Z*-eicosatetraenoyl)-*sn*-glycero-3-phosphocholine	PC 16:0/20:4
1-palmitoyl-2-oleoyl-*sn*-glycero-3-phospho-L-serine (sodium salt)	PS 16:0/18:1
1,2-dilauroyl-*sn*-glycero-3-phospho-L-serine (sodium salt)	PS 12:0/12:0
1-stearoyl-2-arachidonoyl-*sn*-glycero-3-phosphoethanolamine	PE 18:0/20:4
1,2-dilauroyl-*sn*-glycero-3-phosphoethanolamine	PE 12:0/12:0
1-hexadecanoyl-2-(9*Z*-octadecanoyl)-*sn*-glycero-3-phosphoethanolamine	PE 16:0/18:1
1-octadecyl-*sn*-glycero-3-phosphocholine	LPC 18:0
sphingomyelins (bovine brain)	

Table S2. *Cont.*

IUPAC Names of Lipid-Standard Substances	Synonym
1,2,3-tripalmitoyl-glycerol	TG 16:0/16:0/16:0
1,2,3-tristearoyl-glycerol	TG 18:0/18:0/18:0
1,2,3-heptadecanoyl-glycerol	TG 17:0/17:0/17:0
1,3-dioctadecanoyl-2-(9*Z*-octadecanoyl)-*sn*-glycerol	TG 18:0/18:1/18:0
1,3-dioctadecanoyl-2-(9*Z*,12*Z*-octadecadienoyl)-*sn*-glycerol	TG 18:0/18:2/18:0
1,3-dioctadecanoy-l2-(9*Z*,12*Z*,15*Z*-octadecatrienoyl)-*sn*-glycerol	TG 18:0/18:3/18:0
1,2-dilauroyl-*sn*-glycerol	DG 12:0/12:0
1,2-dipalmitoyl-*sn*-glycerol	DG 16:0/16:0
1,2-distearoyl-*sn*-glycerol	DG 18:0/18:0

List of Abbreviations used in Table 3

HETE: hydroxyeicosatetraenoic acid- sn-glycero-3-phosphocholine
HODA: 9-hydroxy-12-oxo-10-dodecenoic acid
HODE: Hydroxyoctadecadienoic acid
HOdiA: 5-hydroxy-6-octenedioic acid
HOOA: 5-hydroxy-8-oxo-6-octenoic acid
HpETE: hydroperoxyeicosatetraenoic acid
HpODE: hydroperoxyoctadecadienoic acid
isoLGD$_2$: isolevuglandine D_2
isoLGE$_2$: isolevuglandine E_2
isoPGA$_2$: isoprostaglandine A_2
isoPGD$_2$: isoprostaglandine D_2
isoPGE$_2$: isoprostaglandine E_2
isoPGF$_{2\alpha}$: isoprostaglandine $F_{2\alpha}$
isoPGG$_2$: isoprostaglandine G_2
isoPGH$_2$: isoprostaglandine H_2
isoPGJ$_2$: isoprostaglandine J_2
isoTxA$_2$: isothromboxane A_2
isoTxB$_2$: isothromboxane B_2
KHdiA: 3-keto-4-hexendioic acid
KOdiA: 5-keto-6-octendioic acid
EC: 5,6-epoxyprostane A_2
EI: 5,6-epoxyprostane E_2
G: glutaric acid
ON: 9'-oxo-nonanoic acid
OV: 5'-oxo-valeric acid
